# Melatonin Regulates Aging and Neurodegeneration through Energy Metabolism, Epigenetics, Autophagy and Circadian Rhythm Pathways

**DOI:** 10.3390/ijms150916848

**Published:** 2014-09-22

**Authors:** Anorut Jenwitheesuk, Chutikorn Nopparat, Sujira Mukda, Prapimpun Wongchitrat, Piyarat Govitrapong

**Affiliations:** 1 Research Center for Neuroscience, Institute of Molecular Biosciences, Mahidol University, Salaya, Nakornpathom 73170, Thailand; E-Mails: anorutj@gmail.com (A.J.); chukorn.nop@mahidol.ac.th (C.N.); sujira.muk@mahidol.ac.th (S.M.); 2 Center for Innovation Development and Technology Transfer, Faculty of Medical Technology, Mahidol University, Salaya, Nakornpathom 73170, Thailand; E-Mail: prapimpun.won@mahidol.ac.th; 3 Center for Neuroscience and Department of Pharmacology, Faculty of Science, Mahidol University, Rama VI Road, Bangkok 10400, Thailand

**Keywords:** melatonin, brain aging, energy metabolism, epigenetics, autophagy, circadian rhythm, neurodegeneration, sirtuins

## Abstract

Brain aging is linked to certain types of neurodegenerative diseases and identifying new therapeutic targets has become critical. Melatonin, a pineal hormone, associates with molecules and signaling pathways that sense and influence energy metabolism, autophagy, and circadian rhythms, including insulin-like growth factor 1 (IGF-1), Forkhead box O (FoxOs), sirtuins and mammalian target of rapamycin (mTOR) signaling pathways. This review summarizes the current understanding of how melatonin, together with molecular, cellular and systemic energy metabolisms, regulates epigenetic processes in the neurons. This information will lead to a greater understanding of molecular epigenetic aging of the brain and anti-aging mechanisms to increase lifespan under healthy conditions.

## 1. Introduction

Understanding the underlying mechanisms of age-dependent alterations in brain structure and functions has become critical for identifying new therapeutic targets and for developing multimodal health-care strategies that meet the needs of an aging population. The pathological processes in the aging brain are associated with molecules and signaling pathways that sense and influence energy metabolism, e.g., insulin, insulin-like growth factor 1 (IGF-1), Forkhead box O (FoxOs), sirtuins (SIRT), autophagy, circadian rhythms and mammalian target of rapamycin (mTOR) signaling pathways. Melatonin, a hormone primarily secreted by the pineal gland, is synthesized from tryptophan under the control of numerous enzymes that are inhibited or stimulated by the light/dark cycle [1,2]. Nocturnal melatonin is a signal to mediate the circadian message for the entire body. Pineal melatonin displays a circadian pattern driven by primary circadian clock signals from the suprachiasmatic nucleus (SCN).

Melatonin not only associates with the energy metabolism pathways but also regulates epigenetic processes in neuronal cells. Energy metabolism is a vital modulator of epigenetic processes of the neural system. Melatonin associates with molecules and signaling pathways that sense and influence energy metabolism, including insulin/IGF-1 [3,4], FoxO and sirtuin pathways [5,6,7]. These pathways are now implicated in the epigenetic processes of both young and aging brains and associated with neurodegenerative diseases. The effects of melatonin in regulating energy metabolism, modulating epigenetics in normal brain aging and neuropathological aging will be discussed. An understanding of how neuronal cells are influenced by energy availability will explain the complex nature of the aging brain in both normal and diseased states.

## 2. Brain Energy Metabolism

### 2.1. Insulin/IGF-1 (Insulin-Like Growth Factor 1) Signaling Pathways and Brain Energy

Neurons require energy to support action potentials, neuronal plasticity and neurotransmission; thus, age-related neuronal energy deficits contribute to the cognitive decline and to the pathogenesis of several neurodegenerative disorders [8]. Insulin/IGF-1 signaling pathways establish a complicated signaling network with close connections to mitochondrial bioenergetics, biogenesis and redox homoeostasis [9,10]. Insulin and IGF-1 bind to their receptors on the cell surface, leading to the phosphorylation of tyrosine residues on the insulin receptor and the IR substrate. Then, IGF-1 further regulates many cascade pathways. For example, IGF-1 activates phosphoinositide 3 kinase-protein kinase B (PI3K–Akt) signaling and then inactivates FoxO transcription factors [11].

Although a decrease in the activation of the insulin/IGF-1 pathway seems to extend the longevity, normal brain functions require a normal insulin/IGF-1 signaling cascade [12,13]. This pathway regulates synaptic plasticity and neuronal survival via the maintenance of neuronal mitochondria during aging [10]. Ames dwarf mice showed a longer lifespan and normal cognitive function in advanced age. These mice showed very low growth hormone (GH) level and undetectable IGF-1 in circulation but increased hippocampal GH and IGF-1 protein levels as compared with the wild type. Increased phosphorylation by Akt and cyclic AMP responsive element-binding protein (CREB) were also detected in the hippocampus of Ames dwarf mice. These features might contribute to the maintenance of cognitive function during aging [14,15]. In addition, over-activated insulin and PI3K/Akt pathways were associated with many pathology characteristics of Alzheimer’s disease (AD) [16,17].

Accumulating evidence suggests that low levels of circulating IGF-1 and impairments of insulin/IGF-1 signaling in the brain contributed to age-dependent cognitive decline, such as Alzheimer’s disease [18,19]. As seen in the Rotterdam Study, which surveyed 1014 persons for the prevalence of dementia, a higher level of IGF-1 receptor stimulating activity was associated with a higher prevalence of dementia [8,20]. In addition, the insulin resistance observed in diabetes constitutes a risk factor for Alzheimer’s disease [21,22]. On the other hand, the insulin/IGF-1 system is involved in many protective pathways. For example, the PI3K/Akt pathway entails phosphorylating FoxO transcription factors, resulting in shuttling phosphorylated FoxO from the nucleus to the cytosol, thereby preventing the transcription of FoxO-driven pro-apoptotic genes [23,24]. Additionally, the anti-apoptotic effect of PI3K–Akt signaling is a consequence of the phosphorylation and inhibition of glycogen synthase kinase 3 β (GSK3β), which, in its active form, phosphorylates anti-apoptotic Bcl-2 and Bcl-xL. Insulin prevents cytochrome c release in the perfused brain in a PI3K-dependent pathway. Insulin increases the total and surface expression of glutamate transporter in astrocytes by a pathway involving the PI3K–Akt/mTOR signaling cascade [25].

### 2.2. Melatonin and Metabolic Pathways

Melatonin (*N*-acetyl-5-methoxytryptamine) is a molecule that is secreted by the pineal gland and that can also be produced in the retina, extraorbital lacrimal gland, Harderian gland, gastrointestinal tract, blood platelets, and bone marrow cells [1,2,26]. Multiple actions of melatonin include: (i) G-protein-coupled melatonin receptors signaling cascade; (ii) inducing QR2; (iii) destroying reactive oxygen and reactive nitrogen species; (iv) increasing calmodulin degradation; (v) binding to nuclear receptors to alter the transcription of target genes; and (vi) modulating hemopoiesis and immune cell production and function [27,28,29].

Melatonin is involved in energy expenditure and body weight regulation. In pinealectomized rats, after an increase in body weight and exogenous melatonin supplement, reverse body weight gain occurs. Similar effects on reduced body weight and visceral fat were observed in both young and middle-aged rats. Additionally, this effect on decreased weight gain can be found in animals fed either a high-fat diet or high fructose [30,31]. The melatonin receptor seems to play a role in obesity. Selective agonists of melatonin receptor type 1 (MT1) and melatonin receptor type 2 (MT2), piromelatine (NEU-P11) and ramelteon, had similar effects on decreasing body weight and blood pressure, which were similar to melatonin-induced effects [32].

Pinealectomy caused a lack of melatonin in rats, which displayed reduced insulin sensitivity and reduced GLUT4 gene expression [33]. In humans, melatonin may improve metabolic syndrome via its anti-hyperlipidemic action. Melatonin inhibits insulin release through MT1 and MT2, which are expressed in pancreatic β-cells [34]. Altered plasma melatonin rhythms in weight-matched type 2 diabetes and non-diabetic individuals support a possible role of melatonin in type 2 diabetes etiology [35,36].

Melatonin is associated with the sensing processes for metabolic status by the primary pathways involved with the insulin/IGF-1 pathway [37]. The cellular energetic state is believed to respond through the activation of different mechanisms, with the best-known mechanism involving NAD^+^-dependent deacetylases, which are called sirtuins [38,39]. This mechanism will be introduced and discussed in Section 2.4.

Insulin, growth hormone (GH) and IGF-1, integrate many physiological responses during aging. Reducing activation of the PI3-K/Akt signal can extend lifespan in organisms from yeast to mammals. In *Caenorhabditis elegans*, genes known to be involved in the insulin/IGF-I pathway include *dauer-constitutive-2* (*daf-2*), which is a homolog of insulin/IGF-1-like receptor, as well as *daf-16* (Forkhead transcription factor) and *daf-18* (PTEN, Phosphatase and tensin homolog). Mutations in these factors result in increased or decreased lifespan [40,41,42,43].

Mammalian models with reduced GH and/or IGF-1 signaling increase longevity compared with intact animals. Mutant mice with anterior pituitary dysfunctions, such as Snell (defect in the pituitary specific transcription factor-1 gene (*Pit-1*)) and Ames dwarf mice (recessive point mutation in the prophet of *Pit1* (*Prop-1*) or paired-like homeodomain transcription factor in *Prop-1*), show dwarf characteristics, female infertility and severely low insulin, IGF-1, glucose, and thyroid hormone level. Interestingly, these mice have a greater than 40% increase in lifespan. The prolonged lifespan effects were also found in mice with the defect in IGF-1 or in the IGF-1 receptor and in *lit/lit* mice, which have a mutated GH-releasing hormone receptor. On the other hand, GH transgenic mice have early puberty, elevated IGF-1 and insulin levels and develop insulin resistance. The lifespan of these mice is significantly shorter compared with the wild-type animals [44,45,46].

Melatonin has been implicated in obesity and in the regulation of insulin activities. Studies in pinealectomized animals induced insulin resistance and glucose intolerance in type 2 diabetic rats [3,47]. Melatonin influences MT1- and MT2-receptor-mediated insulin secretion both *in vivo* and *in vitro*. Melatonin displays a protective effect against reactive oxygen species (ROS) generation in pancreatic β-cells, which are easily susceptible to oxidative stress. The plasma melatonin level and arylalkylamine-*N*-acetyltransferase activity are lower in diabetic rats than in nondiabetic rats. In contrast, arylalkylamine-*N*-acetyltransferase mRNA increased, and the insulin receptor mRNA decreased in the pineal gland, which indicated a close relation between insulin and melatonin [34,41,48]. Melatonin is associated with chronic inflammation in obesity. Obese animals have higher serum levels of interleukin-17 (IL-17). Insulin and IGF-1 increase IL-17-induced expression of inflammatory chemokines/cytokines via a GSK3β dependent pathway, which is inhibited by melatonin via suppression of Akt activation [49].

Melatonin injections resulted in increased circulating GH levels and increased serum IGF-I levels, concomitantly lowering somatostatin levels. These results are associated with significantly decreased hypothalamic norepinephrine turnover [4,50]. The GH rhythm was suppressed in pinealectomized rats; after melatonin replacement in these rats, GH and IGF-1 levels increased during the day [3].

The effects of melatonin on PI3k/Akt signaling in peripheral tissues compared with the brain are different and depend on the stress or injury model. For instance, melatonin showed the protective effects against brain injury by activating Akt and its downstream targets in a middle cerebral artery occlusion model [51] and a kainic acid-induced hippocampal excitotoxicity model [52]. In aged neuronal cell culture, melatonin increased Akt activation, subsequently leading to GSK3β inhibition and an increase in FoxO1 phosphorylation [53]. The effects of melatonin and PI3K–Akt activities in the brain still need to be further explored.

### 2.3. Epigenetics and Aging

Epigenetic processes regulate gene expression caused by modifications at the chromatin level without modifying the DNA sequence. These novel studies represent a bridge between environments, aging and individual genetic backgrounds. Epigenetic are currently considered part of age-related neuropathological phenotypes [54,55,56].

DNA methylation is the process that silences DNA sequences. During aging, 5-methyl-cytosine distribution is found changing across the genome and leading to decreased global DNA methylation. However, some promoters become hypermethylated [56]. DNA methylation and DNA hydroxymethylation, which is the oxidized form of DNA methylation, are also the centers of interest in epigenetic studies of Alzheimer’s disease. Global levels of DNA methylation and DNA hydroxymethylation positively correlate with markers of Alzheimer's disease, including amyloid beta (Aβ), tau, and ubiquitin expression [57].

Histone acetyltransferase (HAT) and histone deacetylases (HDACs) are the posttranslational modifiers of histones. Histone acetylation is catalyzed by HATs, whereas deacetylation is catalyzed by HDACs. Several different families of HATs and HDACs have been identified. Eighteen HDAC enzymes have been identified in humans and have been categorized into 4 classes, including class I, II, III, and IV. Class I, II, and IV members are zinc-dependent enzymes, whereas the class III family includes nicotinamide adenine dinucleotide (NAD^+^)-dependent enzymes. The sirtuins family belongs to this class.

Aging disrupts the epigenetic processes involved with synaptic plasticity and memory in the hippocampus [58]. Epigenetic features during aging, such as lower HDACs activity in the hippocampus [59], are concomitant with higher chromatin repression, such as di-methyl and tri-methyl histone H3K9 in aging brain [60]. In addition, the senescence mouse model studies have demonstrated epigenetic variation in an age-dependent manner. In senescence-prone mice, learning and memory deficits are associated with losses of monomethyl histone H4K20 and tri-methyl H3K36, which are known to facilitate transcription in the hippocampus. When senescence-accelerated mouse prone 8 (SAMP8) mice are compared with age-matched senescence-accelerated-resistant mouse (SAMR1) mice, many methylated histone modifications changed were found in SAMP8 such as methylated H4K20, H3K27, H3K36 and di-methylated H3K79 [61]. Global histone H3 acetylation levels were reduced in SAMP8 mice compared with control SAMR1 mice [62].

The links between histone acetylation dynamics and hippocampus-dependent memory are emphasized by the effects of histone deacetylase inhibitor administration. Direct infusion of triclosan A into the CA1 layer of hippocampus can interrupt the memory system in young mice but not in aged mice [63]. EVX001688, which is a long-lasting histone acetylation enhancer, increased histone acetylation levels during training in a contextual fear conditioning task in young rats but showed no effect on performance in aging [64].

CREB-binding protein (CBP) and p300 have HAT activity. The CBP/p300 complex has the highest level in the brain and relatively high levels in the lung, spleen, and heart. CBP and p300 are relatively stable in the hippocampus with advancing age [65]. However, this complex may play an important role in developing processes because p300 and CBP are highly expressed in the brains and livers of fetal and newborn mice [66]. CBP and p300 are involved in memory consolidation processes. While spatial memory is being consolidated in the rat dorsal hippocampus, an increase in HAT activity, together with global increases in CBP, p300, and PCAF expression, can enhance the memory task [67].

Insulin and IGF-1 are associated with epigenetic variations in the brain. Valproic acid, which is a histone deacetylase inhibitor, induces weight gain and increases the risk of insulin resistance. This drug regulated the expression of adipokine genes in hypothalamic neurons via modulating the activity of the CCAAT enhancer-binding protein alpha (CEBPα) [68].

Age-dependent metabolic syndrome is a risk factor for impaired cognition and for Alzheimer’s disease. Altered DNA methylation and insulin resistance in the brain are associated with pathogenesis from soluble Aβ in Alzheimer’s disease [69]. IGF-1 provides neuroprotective effects via action against HDAC1 and HDAC3. HDAC1 expression is upregulated in the brains of the Huntington disease model and the Ca^2+^/calmodulin-dependent protein kinase (CaMK)/p25 double-transgenic model of tauopathic degeneration. The effect of HDAC1 can be inhibited by IGF-1 expression, Akt expression, or GSK3β inhibition [70]. The epigenetic mechanisms due to cellular energy pathway should be further studied in order to elucidate age-related neuropathogenesis.

### 2.4. Sirtuins

The yeast silent information regulator gene and its mammalian homologs, sirtuins, are the centers of interests in aging research. Seven sirtuins (SIRT1–SIRT7) are found in mammals. Sirtuin1 (SIRT1) regulates epigenetic, DNA repair, aging, and programmed cell death and defends against neurodegenerative diseases. The longevity effects of SIRT1 are expected to rely on its enzymatic deacetylation activity on histone and non-histone substrates (more details in a later section).

Sirtuin is the HDAC that requires NAD as the regulator and co-factor for enzymatic activity. NAD is one of the electron transport chain factors and plays an important role in regulating cellular energy. Nicotinamide phosphoribosyltransferase (NAMPT) and nicotinamide/nicotinic acid mononucleotide adenylyltransferase (NMNAT*)* are the key enzymes for NAD biosynthesis. NAMPT is the rate-limiting enzyme in NAD biosynthesis, whereas NMNAT completes NAD biosynthesis by transferring adenine from ATP to NMN [71]. SIRT1 and the NAD pathway play a role in linking cellular energy with aging. Intracellular NAD^+^ levels and the NAD:NADH ratio in the heart, lung, liver and kidney of female Wistar rats decline in middle-aged rats (12 months old) compared with young (3 months old) rats. Decreases in SIRT1 activity and increased acetylated p53 levels were observed in a variety of organ tissues in parallel with a decrease in NAD^+^ levels [72]. A connection was found between NAD biosynthesis and sirtuins associated with the aging process. Depleting cellular NAD^+^ stores attenuates SIRT1 deacetylase activity, leading to many effects, such as SIRT1 regulation of p53 and some apoptotic factors. This change resulted in increased cell death via apoptotic mechanisms [72,73]. SIRT1-mediated deacetylation can bind several transcription factors and cofactors, including FoxO transcription factors, p300/CBP-associated factor and peroxisome proliferator-activated receptor gamma (PPAR-γ) [71].

SIRT1 is expressed in the brain, with high expression levels in the cortex, hippocampus, cerebellum and hypothalamus but low expression in white matter [74]. SIRT1 is abundantly expressed in several areas, particularly in arcuate, paraventricular, ventro- and dorsomedial hypothalamic nuclei [75,76]. These hypothalamic areas regulate food intake and energy expenditure that link SIRT1 to metabolic status. SIRT1 protein in hypothalamus was high after feeding and low during fasted condition [75,77]. In addition, SIRT1 is associated with hormones and neuropeptides that regulate food intake such as leptin [78] and neuropeptide Y/Agouti-related peptide [79]. Further study of SIRT1 in the hypothalamic system will lead to a greater understanding of how caloric restriction (CR) extends lifespan and neuroprotection during aging.

### 2.5. Forkhead Box O (FoxO)

This sirtuin1 targets that are closely linked with insulin/IGF-1 and with energy homeostasis are Forkhead box O (FoxO) transcription factors. The FoxO family has four members, namely, FoxO1, FoxO3, FoxO4, and FoxO6 [11]. FoxOs are regulated by the insulin signaling pathway and have been implicated in regulating metabolism, cellular proliferation, tumorigenesis, the stress response [80], apoptosis [23], neurogenesis [81] and benefit effects of caloric restriction [46,82]. FoxO1 activation in peripheral tissue interferes with gluconeogenesis and with carbohydrate/lipid pathways [83]. Insulin-PI3K-FoxO3 signaling is required for circadian rhythm (for details, see Section 4) in the liver via regulation of *Clock* in PI3K- and FoxO3-dependent manners [84]. In the brain, SIRT1 and FoxO1 controls food intake through transcriptional regulation of the orexigenic neuropeptide Y, agouti-related protein [75,77].

FoxOs link insulin/IGF-1, SIRT1 and hippocampal functions [85]. FoxO6 is highly enriched in the adult hippocampus and is required for memory consolidation. FoxO6-deficient mice display normal learning but impaired memory consolidation in contextual fear conditioning and in novel object recognition [86]. FoxO is fundamental in the pathogenesis of neurodegeneration, such as in FoxO effects on the oxidative stress response upon manganese-induced Parkinson’s disease [87]. FoxO3 contributes to apoptosis, to β-amyloid-induced neuron death [88], and to the accumulation of α-synuclein, which controls the fate of dopaminergic neurons in the substantia nigra [89].

### 2.6. Melatonin and Epigenetics

Epigenetic actions of melatonin that relates to brain aging and neurodegenerative diseases remain poorly characterized. Since both cellular senescence and cancer cell development involve epigenetic alterations, understanding the epigenetic mechanisms of anti-cancer properties of melatonin may provide the explanation for brain aging-related conditions.

Melatonin causes epigenetic effects against cancer cells by modulating both DNA methylation and histone acetylation pathways. Melatonin has been expected to epigenetically affect DNA methylation in breast cancer; however, the mechanism is unclear [90,91]. Melatonin-treated MCF-7 cells show an inverse correlation with DNA methylation levels and with alterations in oncogenic genes EGR3 and POU4F2/Brn-3b, whereas the tumor suppressor gene GPC3 was upregulated by melatonin [92]. The hypermethylation of the CpG island in the promoter region of the MT1 receptor inversely correlated with its expression in oral squamous cell carcinoma [93].

Recent reports have indicated that melatonin has an effect on histone modification. Melatonin can restore liver histone deacetylase, DNA methyltransferase activity, and DNA methylation [94]. Prenatal dexamethasone exposure unregulated HDAC1 expression in the kidneys of offspring. Maternal melatonin co-therapy with dexamethasone attenuated prenatal-induced hypertension by restoring nephron numbers and by modulating HDAC-1, HDAC-2, and HDAC-8 [95]. When neural stem cells are treated with melatonin, significantly increased histone H3 acetylation and enhanced HDAC isoforms were observed as compensatory mechanisms after melatonin-induced histone hyperacetylation. This epigenetic effect of melatonin acts via the MT1 receptor [96,97].

p300 is abundantly expressed in cancer cells, and p300 over expression enhances cyclooxygenase-2 (COX-2) activation induced by diverse proinflammatory mediators. Melatonin significantly suppressed the proliferation of human MDA-MB-361 breast cancer cells and induced apoptosis in a dose-dependent manner. This melatonin-suppressed proliferation was accompanied with inhibited COX-2, p300, and nuclear factor kappa-light-chain-enhancer of activated B cells (NF-κB) signaling. Melatonin inhibits p300 HAT activity and p300-mediated NF-κB acetylation, thereby blocking NF-κB binding and p300 recruitment to the COX-2 promoter [98]. Melatonin also suppresses p300 histone acetyltransferase activity and p300-mediated NF-κB acetylation in the human vascular smooth muscle cell line CRL1999 [99]. The epigenetic effect of melatonin may correlate with nuclear factor erythroid 2-related factor 2 (Nrf2). Melatonin exhibits an anti-inflammatory effect by suppressing pNF-κB but promoting Nrf2 expression [100,101,102]. The CBP/p300 complex directly acetylates Nrf2 in response to arsenite-induced stress. This acetylation leads to increased promoter-specific DNA binding of Nrf2 and establishes acetylation as a novel regulatory mechanism that modulates the Nrf2-dependent antioxidant response. Nrf2-dependent antioxidant enzyme expression is also dependent on Nrf2 acetylation by CBP/p300 machinery [103,104].

### 2.7. Melatonin and Sirtuin System

SIRT1 has been shown to link with aging and cancer. During aging, SIRT1 expression and its activity declined while it is highly expressed in several types of cancer cells. Melatonin protected aging neurons via preserving the relative protein levels of sirtuin1 in SAMP8 mice [5,6,7] and in hippocampus of total sleep-deprived rats [105] but decreased overexpressed sirtuin1 in cancer such as prostate cancer [106,107] and human osteosarcoma [108]. The supportive effect of melatonin on sirtuin1 may act via the NAD system. Melatonin acts as an effective antioxidant to preserve NAD levels under oxidative stress [109]. Melatonin also indirectly regulates SIRT1 expression involved with SIRT1 targets, e.g., p53 [5,6]. Melatonin prevents the activation of ataxia telangiectasia muted, which is an enzyme involved in p53-related apoptotic pathway activation. Moreover, melatonin treatment of neuronal cell cultures also decreases E2F-1, which is a proapoptotic transcription factor [53].

Melatonin may modulate energy homeostasis through the SIRT1-FoxO pathway. The other interesting SIRT1 target is FoxOs. Melatonin may promote FoxOs activities through upregulating SIRT1 and SIRT1 deacetylate FoxOs, leading to transport of this transcription factor to the nucleus. Additionally, melatonin inhibits CREB-binding protein (CBP) and p300 in breast cancer cells in which p300 is expressed at a high level. The cancer cell proliferation was accompanied by significant inhibition of p300 histone acetyltransferase activity and p300-mediated NF-κB acetylation by melatonin, thereby blocking NF-κB binding and p300 recruitment to the COX-2 promoter [98]. The acetylation of FoxOs via CBP and p300 decreases DNA binding and also decrease activities of FoxOs. CBP is a protein that binds to cyclic adenosine monophosphate-regulated enhancer-binding protein, and homologue protein, p300. This complex has the histone acetyltransferase activity [67]. These target genes may act as the feedback regulator in the SIRT1-FoxOs pathway. Since, FoxOs are regulated by the insulin signaling and play important roles in cellular stress response, further studies will clarify the neuroprotective effects of melatonin during the imbalance of energy homeostasis.

## 3. Autophagy

### 3.1. Autophagy and the Aging Process

Autophagy is characterized by the sequestration processes of cytoplasmic material within an autophagosome for degradation by lysosomes. Autophagy acts as a pro-survival mechanism for maintaining normal cellular functions and serves as an adaptive response during various stress conditions, such as amino acid starvation, an unfolded protein response or viral infection. Since the brain requires a lot of energy for action potential generation and other processes, the age-related decline in metabolism contributes to cognitive decline and is a risk factor for neurodegenerative disorders. These diseases may occur when neurons fail to adapt to decreases in basal energy availability [110,111] that correlate with controlling cell homeostasis by the autophagy process. Autophagy is negatively regulated by the mammalian target of rapamycin (mTOR) signaling pathway and by a downstream cascade of autophagy-related proteins (Atg), such as Atg1, Beclin 1 (Atg6), LC3 (Atg8) and Atg5 [112].

The mTOR pathway is another major signaling pathway that affects aging. Activation of this pathway is nutrient-dependent and responds by an energy shift during cellular growth and division. mTOR is a member of the PI3K-related kinase family, which regulates cell growth and proliferation by modulating protein synthesis and transcription. The mTOR complex 1 (mTORC1) consists of mTOR, regulatory associated protein of mTOR (Raptor), LST8/G-protein β-subunit-like protein (mLST8/GbL) and PRAS40. mTORC1 is stimulated by growth promoting conditions but is inhibited by a low nutrient status, growth factor deprivation, stress and the specific inhibitor rapamycin. The TSC1-TSC2 (tuberous sclerosis complex1/2) inhibitory complex is upstream of the mTORC1 pathway. This complex functions as a GTPase activating protein (GAP) for the GTPase Rheb, which is an mTOR activator. The TSC1-TSC2 complex inactivates Rheb to inhibit mTOR signaling. A variety of growth and stress signals regulate mTORC1 signaling through the TSC1-TSC2 complex [113,114]. TORC1 is activated by amino acids, and insulin/IGF-1 is activated through AKT. Activated AKT phosphorylates and inhibits the TSC1-TSC2 complex. The TSC1-TSC complex can also be regulated by AMP-activated kinase (AMPK) and by Ras homolog enriched in brain (RHEB), which binds to and activates TORC1 in a GTP-dependent manner [115,116].

mTORC1 signaling has been shown to influence aging in many organisms. Reduced TOR activity enhances longevity in lower organisms, such as yeast, worms and flies, through higher mammalian animals, such as rodents. mTOR has been strongly linked to the ribosomal protein S6 kinase (S6K), which is a downstream target of TOR. S6K inhibition reduces protein synthesis and extends lifespan in many animal models [117].

Macroautophagy is also a factor that is repressed during aging [118]. When *Atg6*, *Atg7*, and *Atg12*, which are regulators of macroautophagy, are knocked down, the lifespan is reduced in a *C. elegans* model [119,120]. Furthermore, the knockout of *bec-1*, which is an ortholog of the autophagy gene *beclin-1*, suppressed the lifespan in *C. elegans* [119].

The age-related decline in autophagy function may be associated with many age-associated diseases, including neurodegeneration, in normal diet-fed animals and the onset of the disease slowed by caloric restriction treatment [121,122]. Autophagic activity decreases during the course of aging and genes that control brain aging process are strongly associated with lifespan regulation in flies and worms [123]. Autophagy deficiency leads to abnormal accumulation of protein aggregates thus promoting pathological mechanisms associated with neurodegenerative disorders, such as Huntington and Alzheimer’s disease [124,125]. Moreover, the age-induced memory impairment suggests that cognitive function in aging is strongly associated with the autophagic pathway [126]. Many studies support that primary defects in macroautophagy contribute to the pathogenesis of AD. Most studies focus on the relevance of autophagic dysfunction in AD pathogenesis [127] by the intraneuronal aggregates of protein tau, forming neurofibrillary tangles and extra-neuronal β-amyloid senile plaques [128]. In mouse models of AD, reduced IGF-1 signaling protects from disease-associated neuronal loss and behavioral impairment [129]. In addition, lower levels of the autophagy gene *beclin1* have been observed in human aging brains [125]

Several studies have elucidated that CR-linked lifespan extension is dependent on autophagic degradation in *C. elegans* via autophagosome formation. Autophagy genes play a role in reducing mitochondrial respiration or in reducing TOR activity and increasing longevity in mutant nematodes [130]. Unfortunately, the protein modification processes essential to controlling the autophagic process remain unclear. The role of other signaling pathways and target protein modifications, such as acetylation, must also be further studied.

### 3.2. Autophagy and Sirtuin Pathways

Neurodegeneration such as Alzheimer’s disease is closely linked to the metabolic status and numerous reports have demonstrated that SIRT1 and autophagy are correlated with the balance between NAD^+^/NADH for maintaining metabolic homeostasis and cellular survival. SIRT1 may yield the protective effects against AD through modulating autophagy processes. SIRT1 expression could be inhibited by 3-methyladenine (3-MA), which is an autophagy inhibitor. SIRT1 also regulates cellular metabolism through nutrient-sensing pathways, such as AMPK and TOR pathways [131].

AMPK inhibits mTOR and evokes autophagocytosis by resveratrol. Resveratrol binds to phosphodiesterases and triggers cAMP signaling to activate SIRT1. Resveratrol induces autophagy in a SIRT1-dependent manner [132]. An increase in the energetic AMP/ATP ratio activates AMPK, increases NAD^+^ levels and stimulates SIRT [133]. SIRT1 overexpression stimulates the level of autophagy [134]. High calorie diet-fed mice exhibit shorter lifespan, and resveratrol normalizes this effect, suggesting that the AMPK/SIRT1 pathway is involved [135].

The acetylation process plays a significant role in autophagy regulation. The clearance of mutant *huntingtin* through autophagic degradation can be regulated by acetylation at its Lys444 residue [136]. During growth factor deprivation, GSK3 activates acetyltransferase TIP60 through phosphorylating TIP60-Ser86. Activated TIP60 directly acetylates, thereby stimulating the protein kinase ULK1, which is required for autophagy induction [137,138]. Thus, the acetylation of autophagy-related proteins plays an important role in regulating autophagic flux. p300 can acetylate Atg5, Atg7, Atg8 and Atg12 proteins; acetylation by p300 also inhibits autophagy, whereas silencing p300 increases autophagic flux [139]. Atg8 deacetylation is regulated by SIRT1 [140]. Moreover, aggregating misfolded proteins may have an influence on autophagic function. This concept may explain secondary pathological mechanisms in many neurodegenerative diseases. SIRT1 overexpression is also reported to prevent microglia-dependent Aβ toxicity in Alzheimer’s diseases through inhibiting NF-κB signaling by deacetylating the lysine 310 residue of the RelA/p65 subunit of NF-κB, thereby preventing its transcriptional activity [141]. In Parkinsonian models, resveratrol has a protective effect against rotenone-induced apoptosis and enhances α-synuclein degradation. These advantageous properties were shown to occur via autophagy induction [132]. Prolonged treatment with Longevinex, which is a resveratrol derivative, increased autophagy, and this increase correlated with an increase in SIRT1 levels and with FoxO nuclear translocation [142]. This observation suggests that SIRT1-mediated deacetylation of Atg proteins not only stimulates the autophagic uptake of cellular proteins during starvation but also promotes the degradation of damaged organelles to preserve homeostasis.

SIRT1 could promote the expression of components of the autophagy machinery via deacetylation of many transcription factors, which, in turn, activate autophagy genes. The FoxO family members are crucial to this process [143,144]. Under low nutrient conditions, FoxO transcription factors translocate to the nucleus, where these factors activate the expression of genes that are involved in energy metabolism and oxidative stress resistance, as well as of genes implicated in DNA damage repair, cell cycle arrest, apoptosis and autophagy [145,146,147].

FoxO1 activation results in the upregulation of Rab7, which is a small GTPase that mediates autophagosome-lysosome fusion. *Rab7* overexpression stimulates autophagy, whereas *Pab7* silencing inhibits FoxO1-induced autophagy. GTPase is required for mediating FoxO1-induced autophagic flux. In addition, deacetylating FoxO3 by SIRT1 leads to the upregulation of pro-autophagic Bnip3. FoxO3 can increase the expression of multiple autophagy-related genes, such as *ULK2*, *beclin1*, *VPS34*, *Bnip3* and *Bnip3L*, *Atg12*, *Atg4B*, *LC3*, and *GABARAPL1* [145,148,149].

Genotoxic stress can induce autophagy in a p53-dependent process, and p53 can regulate autophagy-inducing genes [150]. A loss of function mutation in the p53 ortholog *cep-1* exhibits a longer lifespan in *C. elegans* by inducing autophagy [151]. However, p53 deficiency does not enhance the lifespan extension conferred by sir2.1 overexpression. During oxidative stress, SIRT1 blocks the nuclear translocation of p53; therefore, SIRT1 can inhibit the nuclear function of p53, which acts as a transcriptional regulator [152].

### 3.3. Autophagy and Caloric Restriction (CR)

Restricting the calories in diet has been used as a method for increasing both the longevity and quality of life. Caloric restriction (CR) has been found to extend longevity and impacts on age related diseases in yeast, worms, insects and rodents; thus, this regimen has become the center of aging research interest [153,154]. In addition, reduced calorie intake improves memory and cognitive brain functions in aged animals [155,156,157] and decreases risk factors for neurodegenerative diseases [158,159], while high calorie diets increase the risk of neurodegenerative disease [135,160,161]. The longevity and health effects of CR appear to act through many pathways. The possible target pathways, including TOR, AMPK, and sirtuins, can detect changes in specific metabolites, such as amino acids, ATP, and NAD^+^. Further evidence indicated that defective insulin receptor (*daf2* mutant) worms lived longer than control worms [42,46]. Moreover, knockdown of autophagy gene products, including *Atg7* and *Atg12*, was shown to shorten the lifespan of both wild type and *daf2* mutant *C. elegans* [162].

The Ser/Thr protein kinase TOR plays a key role in signaling nutrient limitation in the autophagy pathway [163]. In cells lacking sirtuins, CR can extend lifespan via TOR inhibition [164]. The absence of SIRT1 resulted in mTOR, S6K1, 4EBP1 and S6 phosphorylation. These data indicate a role for SIRT1 in mTORC1 regulation because the mTOR pathway is responsive to nutrient and cellular stress and is downregulated in response to stress signals. Therefore, CR appears to induce both SIRT1 and autophagy. During nutrient starvation, high mTOR activity leads to the phosphorylation of Ulk1 at Ser 757, interfering with the interaction between Ulk1 and AMPK to form a complex with Atg1 for autophagosome assembly [165]. Both SIRT1 activation by resveratrol and CR prolonged the lifespan of *C. elegans* only when these organisms are autophagy competent, and their effects are abolished by silencing *beclin1*. Both *beclin1* knockdown and SIRT1 knockout prevent autophagy induction and reverse resveratrol effects. Therefore, CR and resveratrol require functional SIRT1 to stimulate autophagy and to enhance longevity [166]. Interestingly, silencing SIRT1 eliminated autophagy stimulation by resveratrol and by nutrient deprivation in human cancer cell lines. These data show the same result as seen in CR treatment in *C. elegans*. Mouse embryonic fibroblasts derived from fetuses with homozygous SIRT1 deletions could not activate autophagy during starvation [134].

### 3.4. Autophagy and Neuroinflammation

The NF-κB signaling pathway that plays a vital roles to defense against cell damage and autophagy is regulated by the NF-κB system. Autophagy can stimulate NF-κB-dependent inflammatory responses [167], whereas an increase in autophagy can prevent inflammatory responses [168,169]. However, the role of NF-κB signaling in autophagic degradation is unknown.

NF-κB signaling can repress TNFα-induced autophagy. This suppression was linked to NF-κB activation of mTOR kinase, which is an inhibitor of autophagocytosis [170]. The NF-κB signaling pathway inhibits autophagy in macrophages by downregulating *Atg5* and *beclin1* expression, leading to the promotion of apoptosis and inflammation processes [171]. The formation of this condition is stimulated by NF-κB signaling activation [172,173].

The NF-κB pathway involves the I-kappaB kinase (IKK) complex, which contains IKKα and IKKβ kinases. The mTOR/Raptor complexes in response to TNFα and insulin, are involved with IKKα and IKKβ. The activation of the mTOR/Raptor complex by IKKα was induced by Akt kinase, whereas IKKβ repressed the tuberous sclerosis complex (TSC), which is an mTOR/Raptor suppressor, thereby activating the mTOR kinase [174]. TNFα-activated IKKβ suppressed TSC1 and triggered the mTOR pathway [175,176]. The NF-κB-activating kinases IKKβ and NF-κB-inducing kinase can be selectively degraded by autophagy. Additionally, NF-κB signaling can promote cell survival during the heat shock recovery period via autophagy. To support this notion, the inhibition of NF-κB activation could block the autophagic response and increased cell death after exposure to heat shock stress [177]. HSP90 inhibition with geldanamycin interferes with this interaction and induces the degradation of these proteins by autophagy. Overexpressing SIRT1 protected mice from many types of pathogenic inflammation, such as liver cancer [178]. SIRT1 and SIRT6 repressed a critical driver of inflammation NF-κB, either by deacetylating p65 by SIRT1 or by SIRT6 interacting with histones at NF-κB target genes [179,180]. HIF-1a, which is the SIRT target protein, is involved in a proinflammatory response, and p65, which is related to this transcription factor, can be deacetylated and repressed by SIRT1 and by SIRT2 [181,182]. Histones at NF-κB-regulated genes can also be deacetylated by SIRT6, leading to the repression of gene expression [183].

### 3.5. Melatonin and Autophagy

Melatonin can act as either pro- or anti-autophagy. It depends on the stage of autophagy. In normal physiological condition, autophagy acts as pro-survival to maintain homeostasis of the cells. In this situation, melatonin will help or activate autophagy for cell survival. On the other hand, when cells are exposed to ROS or toxic agents, autophagy (excessive levels) will shift to autophagic cell death. In this state, melatonin exhibits protective effects to inhibit excessive levels of autophagy. Several lines of evidence have suggested that melatonin protects against neuronal cell death from methamphetamine (METH) toxicity [184,185]. In addition, melatonin can protect cells from METH toxicity-induced autophagy overactivation leading to lower autophagic cell death [186]. Some lines of evidence have demonstrated that the interaction between beclin1 and anti-apoptotic Bcl-2 negatively regulates autophagy by blocking an essential protein in the autophagy signaling pathway [187,188]. Under normal conditions, Beclin1 binds to Bcl-2 to form the Bcl-2/Beclin 1 complex; however, this complex dissociates, causing increase autophagy levels, such as when Bcl-2 was activated by an upstream pathway, such as Bcl-2 phosphorylation by c-Jun *N*-terminal kinase 1 activation [189,190]. A novel role of melatonin in protecting against cell death from METH-induced autophagy is to dissociate the Bcl-2/Beclin 1 complex and its upstream cascades that lead to cell death [191]. However, melatonin also correlated with chaperone-mediated autophagy (CMA) signaling to increase protein degradation by inhibiting abnormal forms of their proteins. Melatonin and autophagy work synergistically to promote cell survival by decreasing oxidative stress and by delaying immunosenescence. Some experiments have been designed to study the role of melatonin during immunosuppression. Cyclosporine treatment exhibited increased autophagy during oxidative stress but not during aging, whereas autophagy was suppressed, and LC3-II expression was inhibited by melatonin treatment [192,193]. Melatonin can either induce or inhibit autophagy, depending on cellular requirements and oxidative stress levels. Likewise, the dual functions of autophagy (inducing cell survival or cell death) also require further studies to clarify the regulatory roles of melatonin in the complicated autophagy processes.

## 4. The Circadian System

The circadian clock is an endogenous system that acts as an internal time-keeping device, generating approximately 24 h variation in physiology and behavior. This daily variation is defined as circadian rhythms (circa = about; dies = day) [194]. The hypothalamic suprachiasmatic nucleus (SCN) is the master clock in mammals. Photic information from light is conveyed from the retina via the retinohypothalamic tract and is sent to the SCN. Then, the SCN produces synchronizing signals to control the phases of peripheral clock oscillation [195]. The mechanical clock gene system is formed by a complicated transcription-translation feedback loop. The transcription factor CLOCK (circadian locomotor output cycles kaput) dimerizes with BMAL1 (brain and muscle ARNT-like 1) [196], forming the heterodimer CLOCK:BMAL1, which activates transcription by binding to the E-box (5'-CACGTG-3') and E-box-like promoter sequences [195]. The other CLOCK homologs, such as neuronal PAS (PER, ARNT, SIM) domain protein 2 (NPAS2), dimerize BMAL1, activating transcription and maintaining normal circadian rhythmicity [197,198]. The *Period* (*Per1*, *Per2*, and *Per3*) and *Cryptochrome* (*Cry1* and *Cry2*) genes are the targets of CLOCK:BMAL1 and control this transcription complex. The oligomerization and nuclear translocation of the PER:CRY complex results in the inhibition of CLOCK:BMAL1-mediated transcription [195,199]. Most of the clock genes exhibit 24 h fluctuation changes in the SCN and in peripheral tissues, except for *Clock*. *Clock* does not oscillate in the SCN [200]. Reverse erythroblastosis virus α (Rev-Erbα) is a negative regulator of BMAL1 expression [201], whereas retinoic acid receptor-related orphan receptors α (RORα) and RORγ positively regulate BMAL1 expression [202] via ROR response elements (RORE) [203]. Further regulatory mechanisms include post-translational modification processes, such as acetylation, methylation, phosphorylation and sumoylation. These processes provide additional levels of regulation to sustain and stabilize the accuracy of the circadian oscillation based on the 24-h solar cycle [204].

### 4.1. Melatonin and the Regulation of Clock Genes

Melatonin is primarily secreted at night and is defined as the “hormone of darkness” [2,205,206]. The duration of the nocturnal peak of melatonin secretion also reflects the length of the night [207]. Therefore, the robust rhythms of melatonin are suspected to be a crucial factors for normal circadian function and good health [208].

The expression of melatonin receptors explains the direct action of melatonin in many organs. The presence of melatonin receptors in the SCN and circadian melatonin production represent the association between melatonin production and the circadian rhythm machinery. The light-dark cycle of clock gene expression has been investigated in both melatonin-proficient mice (C3H) and melatonin-deficient mice (C57BL). PER1 (Period 1), CRY2 (Cryptochrome 2), and BMAL1 displayed lower levels in the adrenal cortex of C57BL mice than in C3H mice [209]. In the mouse striatum, pinealectomized mice did not display circadian rhythms of *Per1* mRNA and PER1 protein levels [210]. Primary neuronal cultures derived from the murine striatum demonstrated that melatonin decreased *Per1* and *Clock* expression but increased *NPAS2* expression and showed no effect on the *Bmal1* level. However, these effects were not observed in MT1 knockout animals [211]. A melatonin experiment in hypertensive TGR (mRen2)27 rats yielded a phase-dependent effect on *Per2* and *Bmal1* expression in the heart, particularly during the dark phase, suggesting that melatonin is involved in the clock gene regulatory system; however, the exact mechanisms have not been elucidated [212].

### 4.2. Epigenetic Regulation of Clock Genes

Histone phosphorylation, acetylation, and DNA methylation, which modify circadian *clock* gene expression, have been shown to follow circadian rhythms [213,214,215,216]. DNA methylation in the SCN participates in regulating circadian rhythms. A shorter 22 h life cycle in mice altered global transcription in the SCN and the genome-wide methylation profile, leading to global alterations in promoter DNA methylation. These alterations included areas that contained clock genes and genes involved in synaptogenesis, axon guidance and hormone signaling. Behavioral, transcriptional and DNA methylation changes were reversible after re-entrainment to a 24 h per day cycle. Directly infusing a methyltransferase inhibitor to the SCN can suppress period changes. These data indicated that animals exposed to a 22 h light–dark cycle had long-lasting changes in the SCN transcriptome caused by altered DNA methylation processes [217,218,219]. Histone methyltransferase, MLL1, which methylates histone H3 at lysine 4 (H3-K4), is also associated with CLOCK and is recruited to promoters of CCGs in a circadian manner. H3-K4 methylation at these promoters also displayed rhythmicity and was linked to transcriptional activation [219]. Some reports have demonstrated that rapid phosphorylation of Histone 3 on Serine 10 (H3S10) in the SCN is triggered in response to light. This phosphorylation results in the induction of *Per1* and immediate-early gene expression, such as *c-fos*, indicating that light-mediated signaling regulates circadian gene expression by remodeling chromatin [213]. These findings underscore the involvement of epigenetic mechanisms and circadian regulation.

CLOCK:BMAL1 mediated activation of CCGs has been shown to be coupled to circadian changes by histone acetylation at their promoters [214]. CLOCK also possesses intrinsic histone acetylase activity. Because CLOCK binds to E-box regions of DNA, HAT activity of CLOCK can selectively remodel chromatin at the promoters of CCGs [220]. HAT activity of CLOCK acetylates non-histone substrates, such as BMAL1, leading to facilitated CRY-dependent repression [221]. In addition, the transcription factor CLOCK has intrinsic histone acetyltransferase activity. CLOCK binds to H3K9 and K14 at the promoters of CCG [220]. HAT activity of CLOCK also acetylates non-histone substrates, such as its own binding partner, BMAL1 [221]. CLOCK specifically acetylates BMAL1 at a conserved residue, which enhances CRY-mediated transcriptional repression.

### 4.3. Connection among the Circadian Clock, Epigenetic Variation and Metabolism

Circadian clock genes and metabolic status have a connection. Circadian disruption caused by abnormal circadian melatonin secretion has been proposed to be the cause of obesity development. Hypothalamic obesity is obesity resulting from polyphagia and from increased body weight gain that emerges after extensive suprasellar operations to excise hypothalamic tumors. Patients with hypothalamic obesity display increases in morning and night salivary melatonin compared with controls [222]. Epigenetic marks in circadian rhythm genes are able to modulate metabolic functions. In human studies, rotating shift work has been found to be associated with many components of metabolic syndrome [223]. Long-term shift work results in hypomethylation of CLOCK and hypermethylation of CRY2. Hypoxia inducible factor 1 α (HIF1α) is a part of the master CLOCK gene/protein interaction network that might modulate insulin resistance [224,225].

Another connection between the circadian clock, metabolism, and aging is the interaction between the circadian clock and SIRT1 [226]. SIRT1 is the only HDAC whose enzymatic activity is NAD^+^-dependent; thus, SIRT1 has been directly linked to the control of metabolism and aging [227]. Recently, SIRT1 has been introduced as a critical regulator of the circadian clock machinery [228,229]. The BMAL1:CLOCK complex and the BMAL1:CLOCK:PER2 complex interact with SIRT1. SIRT1 binds to the CLOCK:BMAL1 complex at clock gene promoters and deacetylates BMAL1 at the Lys537 area [228,229,230,231]. In turn, SIRT1 is regulated by the circadian system. The *Nampt* gene is under the direct transcriptional control of the BMAL1:CLOCK complex. The expression of *nampt* and NAD^+^ levels demonstrate circadian oscillation, which suggests circadian control of SIRT1 activity [73,229,231,232]. SIRT1 may participate in this effect because SIRT1 and CLOCK variants have an effect on resistance to body weight loss that could be related to the human chronotype. Participants who carry minor alleles at SIRT1 and CLOCK loci displayed a high resistance to weight loss and a lower weekly weight loss rate than people who have the homozygotes for both major alleles [233]. By increasing the possibility of SIRT1, NAMPT and the circadian clock system in regulating metabolic status may lead to the development of a novel treatment for obesity.

### 4.4. Melatonin, Circadian Clock and Aging

Circadian clock dysfunction contributes to aging and to age-related pathologies. BMAL1-deficient mice develop a premature aging phenotype, characterized by multiple age-related abnormalities and an almost threefold reduction in lifespan [234]. The *Clock^−/−^* mice exhibit an increased rate of inflammation, cataracts and a 15% reduction in longevity [235]. Although the lifespan of *Clock* and *Per2* mutated mice after exposure to non-lethal doses of ionizing irradiation have not been documented, these mice have a shorter lifespan and exhibit some senescence phenotypes [236,237]. In addition to the importance of the circadian clock in accelerating aging, the circadian clock also controls other systems known to be associated with aging, such as the control of metabolism, oxidative stress response, and DNA repair [188,238].

Age-related changes in the SCN may lead to circadian dysfunctions, such as decreased circadian neural activity [239], decreased amplitudes of the circadian body temperature rhythms [240], altered serotonin rhythms in the SCN [241], altered neuropeptide contents and GABAergic networks in the SCN [242], and altered SCN sensitivity [241,243,244]. Melatonin production, amplitude and its pulsatile release from the pineal gland decrease upon aging [245]. Disturbed circadian melatonin rhythm has profound effects on the health and well-being of the elderly subjects [246,247]. *Per2* and *Bmal1* disruption in mice has been compared to some workers with alterations in behavioral rhythms, to the development of malignant tumors, to metabolic syndrome [248,249] and to premature aging [250].

Melatonin receptors are present in the mammalian SCN, and circulating melatonin can reach the central SCN clock. This feedback is important in the long-term functioning of the circadian system, e.g., aging [207]. The presence of MT1 receptors in the SCN indicates that exogenous melatonin can affect circadian regulation. The differential effects of melatonin in restoring daily rhythms of serotonin [241], antioxidant enzymes and lipid peroxidation [243] have previously been reported. Moreover, aging results in differential alterations in daily rhythms of expression of various clock genes (*Per1*, *Per2*, *Cry1*, *Cry2* and *Bmal1*) in the SCN, and the therapeutic effects of melatonin in restoring such age-induced alterations have also been documented. The mRNA expressions of various clock genes in SCN in 3, 12 and 24 months showed that the circadian variation were due to age. In young rats (3 months), *Per1*mRNA expression peaked at zeitgeber time-6 (ZT-6), while *Per2*, *Cry1* and *rCry2* at ZT-12 and *Bmal1* peaked at ZT-18. The phases of circadian mRNA expression exhibited the change of daily rhythms of these genes. Melatonin administration for 11 days restored of the rhythm of *Per2*, *Cry1*, *Cry2* and *Bmal1* in 12-month, whereas, the fluctuation of *Cry1*, *Cry2* and *Bmal1 were* restored at 24 months old. The abolishment of fluctuation of these circadian genes may be due to the decrease of the SCN melatonin receptor number [251]. The difference of melatonin effect on each circadian gene in the progress of aging needs to be further studied.

### 4.5. Circadian Regulation and Autophagy

The diurnal variation in autophagy activities and the number of autophagic vacuoles found, vary during the day in many tissues [252,253,254,255]. The measurement of autophagic markers revealed that autophagy in the liver flux reached a maximal peak during the afternoon and declined to minimum during the dark period [256]. In the retina, autophagy was stimulated by light under constant conditions and obtained maximal responses during the late dark and early light phases [257]. Several genes in the autophagy pathways were expressed in an oscillate manner when exposed to varied nutrient conditions [258,259].

C/EBPβ, which is a leucine zipper transcription factor, plays an important role in linking the circadian rhythm and autophagy gene expression. C/EBPβ is expressed in a rhythmic manner and is regulated by the liver clock. C/EBPβ stimulates autophagy gene expression and induces autophagic protein degradation in cultured hepatocytes. This transcription factor binds directly to the promoter regions of autophagy genes and then activates transcription [260,261]. Several lines of evidence demonstrate the roles of mTOR signaling in circadian clocks. In the SCN, mTOR activity displays robust circadian rhythms, and its rhythms are affected by light cues [262]. A genetic modification increasing mTOR activity displayed abnormal circadian rhythm with a longer period in *Drosophila* [263]. mTOR inhibitor treatment also decreases light-induced PER protein expression and helps modulate the phase shifts in behavior in animals [264]. Disrupting the circadian rhythm leads to many pathological conditions. Increasing the mTOR signaling pathway is associated with accelerated aging. In contrast, the circadian clock is one of the important systems for controlling autophagic activity [259]. Mice lacking *Bmal1* had elevated mTORC1 activity both *in vivo* and in cell culture [265]. Interestingly, the pharmacological inhibition of mTORC1 by rapamycin increased the lifespan of *Bmal1^−/−^*mice by 50%. BMAL1 regulates the mTOR signaling pathway by acting as a negative regulator of mTORC1 signaling. These findings demonstrate the role of the circadian clock in regulating the mTOR signaling pathway in mammals.

### 4.6. Role of Melatonin and SIRT1 as Circadian Modulators in Memory Processing

Memory formation processes contribute to the circadian rhythms in vertebrate and invertebrate models [266]. One of the key problems during aging is memory impairment, which is one of the symptoms of Alzheimer's disease. Melatonin may improve memory processes during aging through SIRT1 and circadian modulation because melatonin increased hippocampal SIRT1 level and improved cognitive functions in total sleep deprivation models [105]. Moreover, memory formation is also controlled by circadian regulation. Evidence from one study indicates that functional clocks are present in many parts in the brain, including the hippocampus, suggesting the presence of an autonomous clock; *Per2* expression was found to be rhythmic in isolated hippocampi [267]. A time-of-day effect is observed in memory formation, thereby linking the circadian clock to this biological process [266]. Long-term potentiation (LTP) in the hippocampus has been demonstrated to undergo circadian changes [268]. Moreover, mitogen-activated protein kinase (MAPK) phosphorylation displays rhythmicity in the hippocampus, and inhibiting this oscillation leads to impairment in the persistence of long-term memory [269].

An important function of the circadian clock is to synchronize different metabolic processes in an organism and to synchronize an organism to its environment to guarantee the optimal performance of different organ systems. The physiological processes controlled by the circadian clock include energy metabolism, sleep-wake cycles, hormone secretion, body temperature, locomotor activity, and visceral organ functions; all these mechanisms exhibit daily variations [270,271,272,273]. In humans, abnormal circadian clock rhythms can be found in individuals performing shift work and are expected to be the cause of neurodegeneration, metabolic syndromes, cardiovascular diseases and cancer [274].

The circadian acetylation function of SIRT1 has been described in aging and in neurodegeneration [275]. SIRT1 was shown to deacetylate and coactivate retinoic acid receptor β (RARβ), which leads to activation of the transcription of *Adam10*, which is a gene that encodes α-secretase [276]. Cleavage of the amyloid precursor protein by α-secretase prevents the production of the toxic amyloid β peptides that cause Alzheimer’s disease. Thus, SIRT1 appears to have a neuroprotective role [276]. SIRT1 also deacetylates tau and prevents tauopathy that is evident in several neurodegenerative diseases [277]. Moreover, treatment with the SIRT1 activator resveratrol or ectopic SIRT1 expression was shown to prevent neuronal cell death [278].

Recent studies have reported that SIRT1 also plays a major role in synaptic plasticity and memory formation. Brain-specific SIRT1 mutant mice or SIRT1 whole-body knockout mice displayed deficits in learning and memory [279,280]. Brain-specific SIRT1 mutant mice exhibited lower levels of CREB protein expression in the hippocampus. CREB expression was found to be downregulated by a microRNA, miR-134. SIRT1 negatively regulates miR-134 expression, thus, in turn, regulating CREB expression. miR-134 overexpression in the hippocampus mimics the loss of SIRT1, whereas knocking down miR-134 in the hippocampus ameliorates memory defects in the SIRT1 mutant mice [65,279].

## 5. Conclusions

**Figure 1:**
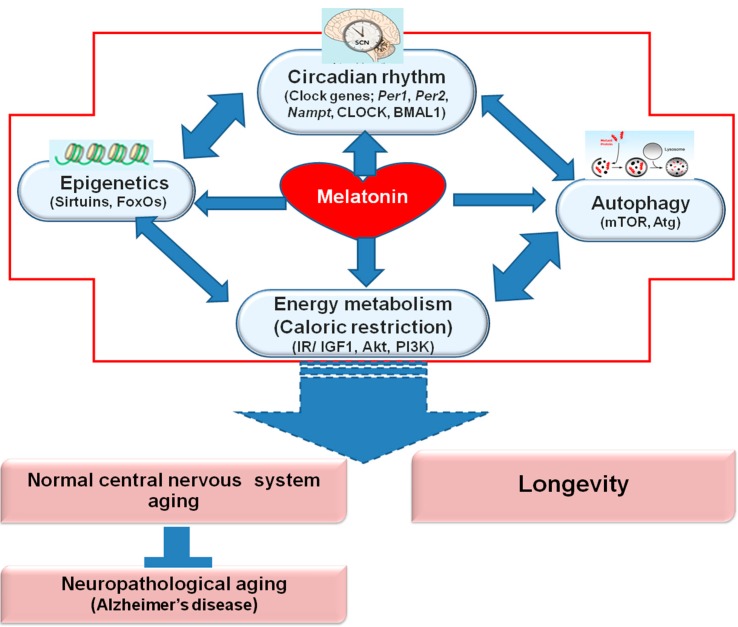
Mechanism of melatonin in controlling normal nervous system aging, neuropathological aging and longevity. The multiple mechanisms of action of melatonin include the following: 1. regulating the circadian rhythm, including several clock genes (*Per1*, *Per2*, *Nampt*, CLOCK, and BMAL1); 2. epigenetics, including sirtuins and FoxOs (forkhead box O); and 3. autophagy, including mTOR (mammalian target of rapamycin) and Atg (autophagy-related proteins). Melatonin regulates several molecules and signaling pathways that sense and influence energy metabolism, including insulin/IGF1, Akt (protein kinase B), and PI3K (phosphoinositide 3 kinase). These pathways regulate normal nervous system aging. Age-related neuronal energy deficits contribute to the pathogenesis of several neurodegenerative disorders, such as Alzheimer’s disease and Parkinson’s disease. The anti-aging properties of melatonin regulate energy metabolism, leading to longevity.

In this review, we have discussed age-related changes in the normal nervous system that occur because of the mechanisms of energy metabolism. Melatonin plays a major role in regulating the following processes: 1. the circadian rhythm, including several clock genes (*Per1*, *Per2*, *Nampt*, CLOCK, and BMAL1); 2. epigenetics, including sirtuins and FoxOs; and 3. autophagy. Melatonin regulates several molecules and signaling pathways that sense and influence energy metabolism, including insulin/IGF1, and PI3K/Akt. These pathways regulate normal nervous system aging. Age-related neuronal energy deficits contribute to the pathogenesis of several neurodegenerative disorders, such as Alzheimer’s disease and Parkinson’s disease. The anti-aging properties of melatonin regulate energy metabolism, leading to longevity (Figure 1). A better understanding molecular aging and anti-aging mechanisms is required to increase lifespan under healthy conditions, particularly to improve cognitive functions.
